# β2GPI exerts an anti-obesity effect in female mice by inhibiting lipogenesis and promoting lipolysis

**DOI:** 10.18632/oncotarget.21536

**Published:** 2017-10-04

**Authors:** Shangwen Dong, Miao Qi, Ying Wang, Liming Chen, James Crofton Weaver, Steven Antony Krilis, Bill Giannakopoulos

**Affiliations:** ^1^ Department of Infectious Diseases, Immunology and Sexual Health and Department of Medicine, St George Hospital, University of New South Wales, New South Wales, Sydney, Australia; ^2^ Department of Cardiothoracic Surgery, Tianjin Medical University General Hospital, Tianjin Medical University, Tianjin, China; ^3^ Laboratory of Hormones and Development (Ministry of Health), Metabolic Hospital and Tianjin Institute of Endocrinology, Tianjin Medical University, Tianjin, China; ^4^ Department of Cardiology, St George Hospital, New South Wales, Sydney, Australia; ^5^ Department of Rheumatology, St George Hospital, New South Wales, Sydney, Australia

**Keywords:** apolipoprotein H, β_2_-glycoprotein I, obesity, sexual dimorphism, lipogenesis

## Abstract

In humans, males compared to females have increased visceral adipose tissue which contributes to their increased risk of early death. Mice display analogous sexual diamorphism whereby females are protected from weight gain when fed a high fat diet compared to males. A role has recently been reported for β_2_-glycoprotein I, an abundant plasma protein, in healthy leanness in humans. In this study we investigated the role of β_2_-glycoprotein I in fat metabolism in male and female mice fed a normal chow or high fat diet. We have made a number of novel insights into factors contributing to sexual diamorphism in obesity. Female wild type mice are protected from obesity when fed a high fat diet due to down regulation of lipogenesis in the visceral adipose tissues. This down regulation is due to β_2_-glycoprotein I as female mice deficient in this protein have increased levels of lipogenesis enzymes in their visceral adipose tissues with an accompanying increase in weight compared to female wild type controls. Understanding female specific regulators of obesity may lead to sex specific anti-obesity therapies to address this major health problem.

## INTRODUCTION

Obesity is a major worldwide health problem. A pooled analysis including 19.2 million individuals from 200 countries between the years of 1975 to 2014 found an increase in the age standardised prevalence of obesity in men from 3.2% of the population in 1975 to 10.8% in 2014, and for women from 6.4% to 14.9%. If such trends continue it is estimated that by 2025 global obesity prevalence will reach 18% in men and surpass 21% in women, whilst severe obesity will surpass 6% in men and 9% in women [1]. In a meta-analysis of over 200 prospective studies from multiple regions found a consistent association between obesity with higher all-cause mortality [2]. In conjunction with the increased availability and consumption of inexpensive, high energy foods, and a decrease in physical activity, leading to net energy storage rather than energy expenditure, other important factors predisposing an individual to an increased risk of developing obesity are multiple genetic susceptibility loci delineated in genome wide association studies, including a number that map near hypothalamic regulators of energy balance [3]. The strongest genomewide association signal lies in introns 1 and 2 of the gene Fat Mass and Obesity Associated variant (FTO), which has been found to repress mitochondrial thermogenesis in adipocyte precursor cells [4].

Starting at puberty, females compared to males have a tendency to accumulate fat in the subcutaneous tissue such as the hips and thighs, whilst there is marked difference in the waist to hip ratio, being consistently greater in males compared to females [5]. Central obesity which reflects visceral adiposity as measured by the waist to hip ratio and is an independent risk factor from overall adiposity (the latter measured by the body mass index), for type 2 diabetes mellitus [6, 7], coronary heart disease [8] and mortality from all causes [9]. This sexual dimorphism in fat distribution may account to some extent why men compared to women, for an identical BMI in the obesity range, have a higher all cause mortality rate [2]. This sexual diamorphism has also been noted in the rodent C57BL/6J strain where female wild type (WT) mice compared to male WT mice were protected from obesity for up to 15 weeks when fed a high fat diet [10].

Beta 2-glycoprotein I (β2GPI) (also known as apolipoprotein H) is an abundant plasma protein that is produced in the liver. It belongs to the complement control protein superfamily of molecules, and it has been shown to be strongly conserved in the evolutionary line, being present in all mammals, but also in birds, fish, and reptiles [11].

Over the past few years a complex association between β2GPI and obesity has been emerging in unbiased, non-hypothesis driven, exploratory, association seeking studies in the context of obesity. It has been noted in studies involving zebra fish, rats, mice [12], pigs [13] and beef cattle [14] that there is upregulation of either β2GPI gene transcription or β2GPI protein expression in the adipose tissue of obese animals compared to tissue from non-obese species controls. This relation has also been noted to hold in humans, where it was noted the plasma levels of β2GPI positively correlates with body fat [15]. β2GPI protein levels have also been noted to be increased in the liver and plasma of type 2 diabetic patients with metabolic syndrome [16]. In a recent genetic study the combination of a certain FTO polymorphism with a certain β2GPI polymorphism conferred a protective effect towards healthy thinness [17]. In another study the level of β2GPI in the adipose tissue in individuals with the pro-obesity FTO polymorphism was upregulated [4]. These studies raise the fascinating question of whether β2GPI has a causal role in modulating obesity. We sought to address this question using β2GPI deficient mice on a C57BL/6J background and comparing them to identically housed and fed gender and age matched wild type C57BL/6J mice.

## RESULTS

### Wild type females fed a high fat diet are protected from obesity which is lost in the absence of β2GPI

**Figure 1:**
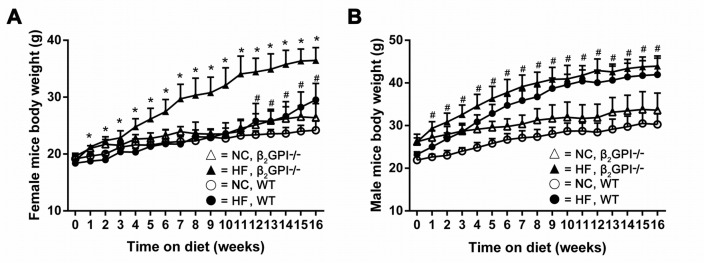
Body weight of β2GPI-/- compared to WT mice recorded on a weekly basis for 16 weeks **(A)** Female; **(B)** male: ○ = NC diet WT; ● = HF diet WT, Δ = NC diet β2GPI-/-, ▲ = HF diet β2GPI-/-. Data are expressed as mean ± SD, n = 4-5 mice per group. ^*^ p < 0.05, significantly greater weight in genotype comparisons; ^#^ p <0.05, significantly greater weight in diet comparisons. NC = normal chow, HF = high fat.

Female β2GPI-/- mice gained significantly more weight than female WT mice when fed a high fat (HF) diet which was observed from week 1 and continued out to week 16 (p < 0.05, Figure 1A). For the first 12 weeks the WT female mice fed a HF diet were the same weight as the WT females that were fed on normal chow (NC). However at weeks 12-16 the WT females fed a HF diet started to gain more weight than the NC fed WT females (Figure 1A). This early anti-obesity phenomenon has previously been reported [10]. However, the body weight of the male β2GPI-/- mice were not significantly different from male WT mice from week 1 to 16 weeks of HF diet (p > 0.05, Figure 1B). The male WT mice fed a HF diet gained significantly more weight from week 1 compared to the male WT mice fed NC diet, (Figure 1B) emphasising the sexual diamorphism of this early anti-obesity phenomenon as previously reported.

### The absence of β2GPI promotes an increase in visceral fat in female mice fed either a normal chow or high fat diet

**Figure 2:**
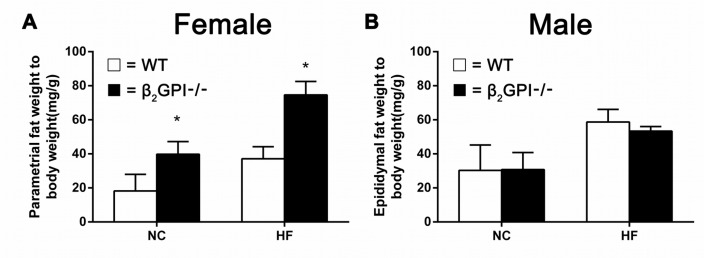
VAT weight in β2GPI-/- mice fed NC or HF diet for 16 weeks **(A)** Female; **(B)** male. □ = WT, ■ = β2GPI-/-. Data are expressed as mean ± SD (n = 4-5 mice per group). ^*^p < 0.05. NC = normal chow, HF = high fat.

We measured the weight of visceral adipose tissue (VAT). In female mice we quantitated the parametrial fat, while for the male mice we quantitated epididymal fat, the major visceral fat depots in female and male rodents respectively. Female β2GPI-/- mice had significantly increased parametrial fat deposits compared to female WT mice fed either NC or a HF diet (p < 0.05, Figure 2A). There was no difference in the quantity of epididymal fat between male β2GPI-/- and diet matched male WT mice (p > 0.05, Figure 2B).

### Female β2GPI-/- mice fed a high fat diet demonstrate difference in adipocyte morphology, macrophage infiltration of VAT and increased plasma CRP

**Figure 3:**
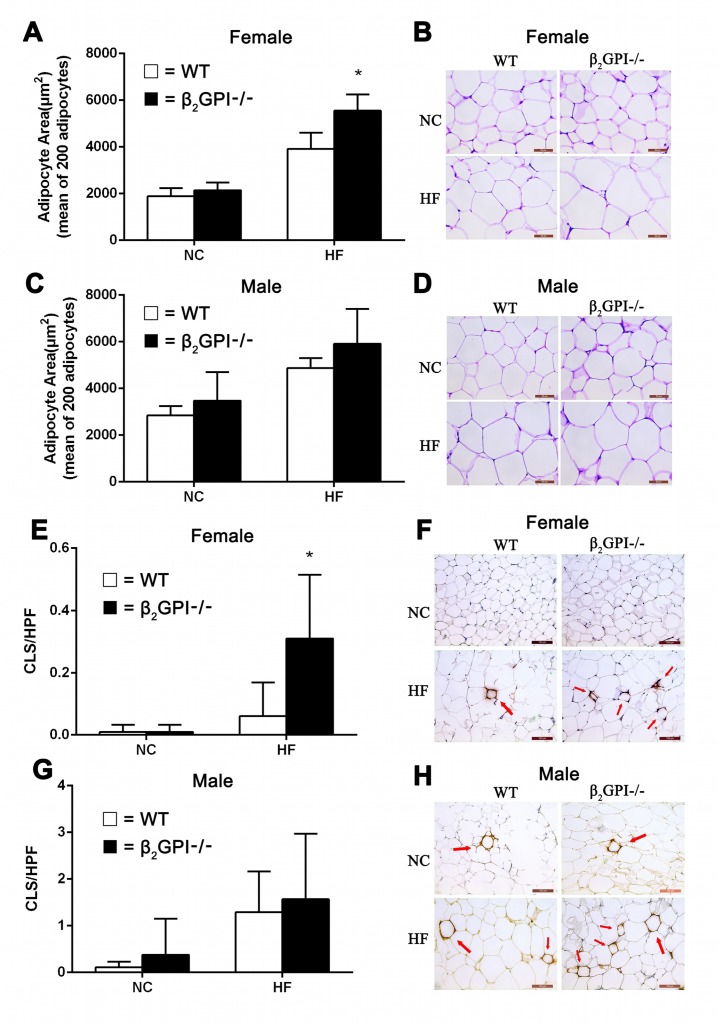
Visceral adipocyte size and infiltration of VAT detected by MAC-2 staining in β2GPI-/- and WT mice fed NC or HF diet for 16 weeks Female **(A, B)** and male **(C, D)** adipocyte area and representative images of adipocyte morphology. Female **(E, F)** and male **(G, H)** crown-like structures (CLS) per high power field and representative images of macrophage infiltration of VAT. Scale bar is 50 μm. Adipocyte area is shown as mean ± SD (n = 5 mice per group, 200 cells per mouse). Number of CLS indicated by arrows in VAT was calculated as numbers per high power field in 20 high-power fields (magnification 200×). ^*^p < 0.05. □ = WT, ■ = β2GPI-/-, NC = normal chow, HF = high fat.

The mean adipocyte area of parametrial/epididymal VAT is shown in Figure 3. Mice fed HF diet show enlargement of adipocytes, moreover, the adipocyte size of female β2GPI-/- mice fed HF diet was significantly larger than female diet matched WT mice (p < 0.05, Figure 3A). Representative images of adipocytes from female and male cohorts are shown in Figure 3B and 3D respectively). There was no difference in the adipocyte size between male β2GPI-/- and male WT mice fed either NC or a HF diet (p > 0.05, Figure 3C). There is increased macrophage infiltration in the VAT of female β2GP-/- mice fed a HF diet compared to female WT mice fed a HF diet (Figure 3E, 3F). There was no difference in macrophage infiltration in the VAT of male β2GPI-/- mice fed a HF or NC diet compared to WT controls (Figure 3G, 3H).

Female β2GPI-/- fed a HF diet have higher plasma C reactive protein (CRP) levels 9.34 ± 0.82 μg/ml, mean ± SD, n = 5 compared to female WT mice fed a HF diet 7.18 ± 0.70, mean ± SD, n = 5, (p < 0.05). This difference was not observed in female β2GPI-/- mice fed a NC diet, 5.12 ± 1.28 μg/ml compared to female WT mice fed a NC diet, 4.95 ± 1.30 μg/ml, mean ± SD, n = 5, p > 0.05.

### β2GPI deficiency regulates lipogenesis enzymes in female VAT

**Figure 4:**
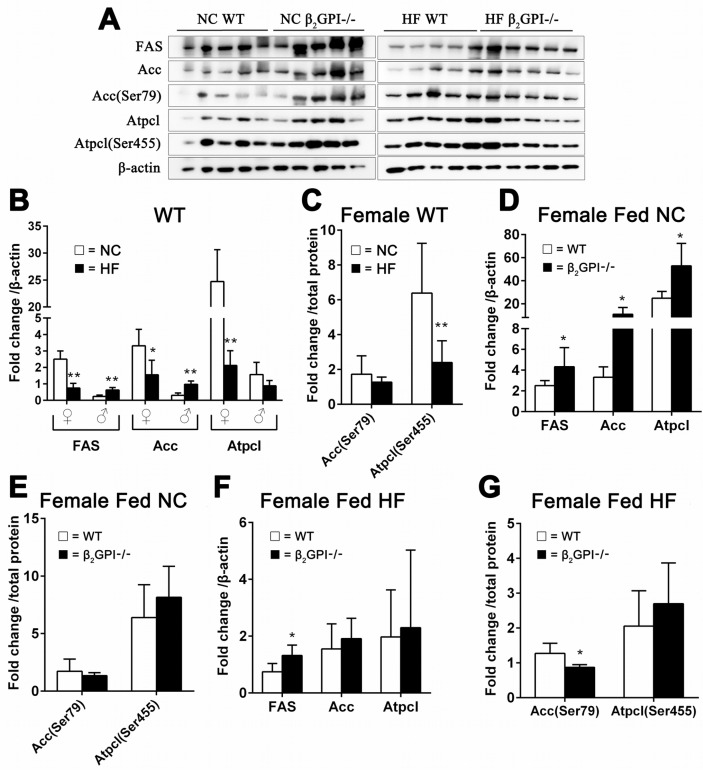
Effect of β2GPI deficiency on VAT lipogenesis enzyme levels **(A)** Western blot analysis of VAT proteins. Quantification of the scanned images normalized to β-actin **(B, D, F)** and to total protein **(C, E, G)**. Panels (B) WT mice fed a HF diet compared to WT mice fed NC. □ = NC, ■ = HF diet. ♀ = female, ♂ = male. (C) Phosphorylation of Acc (Ser79) and Atpcl (Ser 455) as a ratio of the total protein level from (B) for female mice. □ = NC diet, ■ = HF diet. (D) Female β2GPI-/- mice fed a NC diet compared to female WT mice fed a NC diet. □ = WT, ■ = β2GPI-/-. (E) p-Acc (Ser79/Acc) and p-Atpcl (Ser455). (F) Fas Protein, Acc and Atpcl. (G) Phosphorylation of Acc (Ser79) and Atpcl (Ser455) as a ratio to the total protein level from (F). □ = WT, ■ = β2GPI-/-.

ATP citrate lyase (Atpcl) catalyzes the formation of acetyl-CoA from citrate [18]. Phosphorylation of serine (Ser) 455 in Atpcl enhances the catalytic activity of the enzyme and the conversion of citrate to acetyl-CoA [19, 20]. Acetyl-CoA carboxylase (Acc) is responsible for the formation of malonyl-CoA from acetyl-CoA [21]. Phosphorylation of Acc at Ser79 will inhibit its activity [22]. Malonyl-CoA regulates lipid oxidation through the inhibition of the mitochondrial enzyme carnitine palmitoyltransferase-1 and promotes lipogenesis through the provision of a key substrate [23]. A decrease in the activity of Acc inhibits lipogenesis by lowering of cellular malonyl-CoA [22]. Fatty acid synthase (FAS) is a crucial enzyme for de novo lipogenesis by converting malonyl-CoA to fatty acids [18]. When female WT mice were fed a HF diet there was a highly significant down regulation of lipogenesis enzymes compared to female WT mice fed NC (Figure 4A, 4B). In contrast male WT fed a HF diet had a dramatic increase in lipogenesis enzyme expression in VAT compared to male WT fed a NC diet (Figure 4B). There was no difference in phosphorylation of Acc (Ser 79) and Atplc (Ser455) for the male mice (data not shown). In addition there was a significant decrease in the phosphorylation of Atpcl (Ser 455) in the female WT mice fed a HF diet compared to the female WT mice fed NC (Figure 4A, 4C). In the female β2GP-/- mice fed NC there was increased protein expression of FAS, Acc and Atpcl compared to female WT mice fed NC (Figure 4A, 4D), but there was no difference in the p-Acc (Ser79/Acc) and p-Atpcl (Ser455) (Figure 4A, 4E). The lipogenesis enzymes demonstrate an increase in total FAS protein (Figure 4A, 4F) and a decrease in Acc phosphorylation of Ser79 respectively (Figure 4A, 4G) in the female β2GP-/- mice fed a HF diet compared to female WT mice fed a HF diet.

### In both male and female WT mice lipolysis enzymes were inhibited by HF diet. β2GPI had an effect on the levels of a number of lipolysis enzymes in both females and males

**Figure 5:**
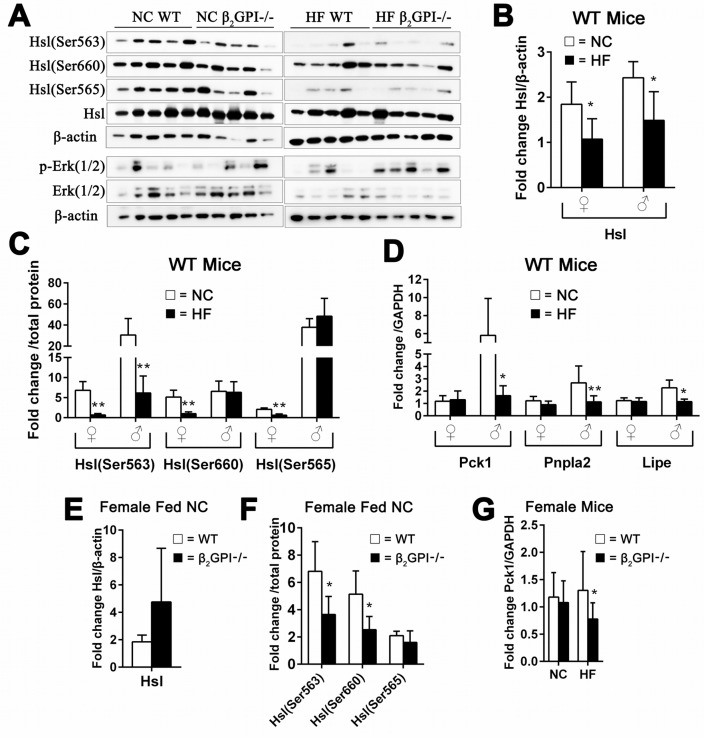
Effect of β2GPI deficiency on VAT lipolysis enzyme activation **(A)** Western blot analysis of lipolysis enzymes in VAT of female mice. **(B)** Hsl – total and **(C)** is phosphorylation of Hsl (Ser563), Hsl (Ser660) and Hsl (Ser565). □ = NC diet, ■ = HF diet. **(D)** mRNA levels of Pck1, Pnpla2 and Lipe. □ = NC diet, ■ = HF diet. ♀ = female, ♂ = male. **(E)** Total Hsl and **(F)** phosphorylated Hsl (Ser563), Hsl (Ser660) and Hsl (Ser565) in female β2GPI-/- and female WT mice fed a NC diet. □ = WT, ■ = β2GPI-/-. (G) mRNA levels for Pck1 in female β2GPI-/- and female WT mice fed a NC and HF diet. □ = WT, ■ = β2GPI-/-.

Lipolysis in VAT is principally regulated by two lipases, adipose triglyceride lipase (Atgl, encoded by Pnpla2), which hydrolyzes the first ester bond of triglycerides, and hormone-sensitive lipase (Hsl, encoded by Lipe), which favors diacylglycerides [24]. Hsl is known to have three major phosphorylation sites. Activated protein kinase A (PKA) phosphorylates Hsl at Ser563 and Ser660, which stimulates Hsl activity [25]. In contrast, AMP-activated protein kinase (AMPK) phosphorylates Hsl at Ser565, which reduces Hsl phosphorylation at Ser563 by PKA and inhibits Hsl activity [25]. Female WT mice fed a HF diet had a significant decrease of Hsl and p-Hsl (Ser563), p-Hsl (Ser660) and p-Hsl (Ser565) compared to female WT fed NC diet (Figure 5A, 5B, 5C). Whereas Pck1 and the lipolysis enzymes Pnpla2 and Lipe were no different (Figure 5D). In the WT male mice fed a HF diet compared to the WT male mice fed a NC diet there was decreased Hsl levels and the p-Hsl (Ser563), whereas there was no change in p-Hsl (Ser660) and p-Hsl (Ser565) (Figure 5B, 5C). Pck1 and the lipolysis enzymes Pnpla2 and Lipe are significantly decreased in male WT mice fed HF diet compared to male WT mice fed NC (Figure 5D).

We found that female β2GPI-/- mice fed NC diet compared to female WT fed NC had decreased phosphorylation of Hsl on Ser563 and Ser660, but had no effect on phosphorylation of Ser565 in VAT (Figure 5E, 5F). Extracellular signal regulated kinase (Erk) has previously been shown to stimulate lipolysis and Hsl [26]. There was no difference in the levels of total and phosphorylated Hsl and Erk (1/2) in the VAT of female β2GPI-/- and female WT mice fed a HF diet (Figure 5A).

Net lipolysis is an equilibrium state between triglyceride breakdown and fatty acid re-esterification. The latter requires glycerol-3-phosphate, the product of glyceroneogenesis, for which the key enzyme is phosphoenolpyruvate carboxykinase (Pepck, encoded by Pck1) [20]. Our results show that female β2GPI-/- mice had markedly decreased expression of Pck1 in VAT when fed a HF diet (Figure 5G). There was no difference in the mRNA levels of the Pnpla2 and Lipe in VAT of female β2GPI-/- and WT mice fed NC or HF diet (data not shown) (p > 0.05).

We examined the lipolysis enzyme Hsl and the phosphorylation at Ser563, 565 and 660, in male NC and HF fed mice. There was no significant difference between diet matched male β2GPI-/- and WT mice, p ≥ 0.05 (data not shown). But there was a decrease in the mRNA levels of Pnpla2 (1.32 ± 0.45, mean ± SD, n = 5, p < 0.01,) in the β2GPI-/- male mice compared to the WT males fed a NC diet (2.67 ± 1.37, mean ± SD, n = 5). There was also a decrease in the mRNA level of Lipe in β2GPI-/- male mice fed a HF diet (0.81 ± 0.07, mean ± SD, n = 5, p < 0.01) compared to male WT mice fed a HF diet (1.15 ± 0.21, mean ± SD, n = 5).

### Female β2GPI-/- mice fed a NC diet demonstrate a decreased food intake

**Figure 6:**
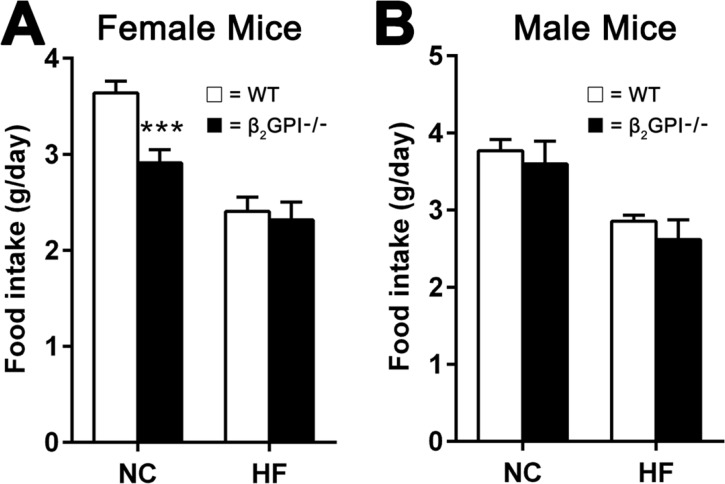
Daily food intake per mouse when fed NC or HF diet for 16 weeks **(A)** Female; **(B)** male, □ = WT, ■ = β2GPI-/-. Data are expressed as mean ± SD, n = 4-5 mice per group. ^***^p < 0.001. NC = normal chow, HF = high fat.

Food intake in female β2GPI-/- mice, compared to female WT mice, was significantly reduced when fed a NC diet (Figure 6A). However no difference in food intake was observed when female mice were given a HF diet (Figure 6A). There was no difference in the male WT and male β2GPI-/- mice fed either a NC or HF diet (Figure 6B).

### Circulating leptin levels in female mice

**Figure 7:**
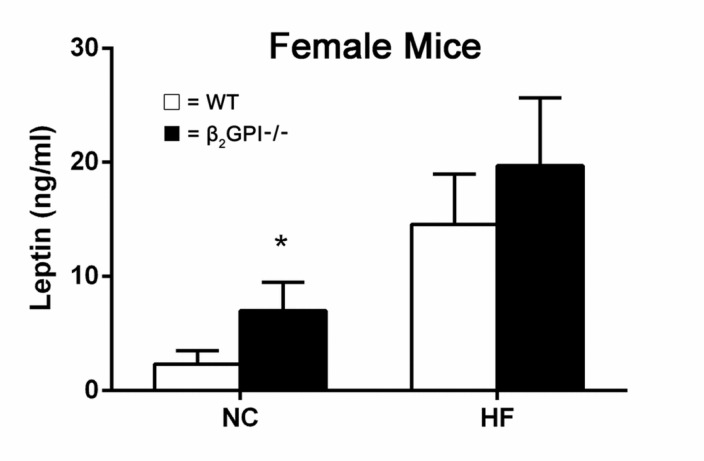
Plasma leptin levels in female β2GPI-/- and WT mice fed NC or HF diet □ = WT, ■ = β2GPI-/-. Data are expressed as mean ± SD (n=5 mice per group). ^*^p < 0.05. NC = normal chow, HF = high fat.

Female β2GPI-/- mice had an increased plasma leptin level compared to female WT mice fed a NC diet (p < 0.05) (Figure 7). There was a trend showing an increase of leptin levels in female β2GPI-/- mice fed a HF diet compared to WT females fed a high fat diet, but it did not reach statistical significance (Figure 7).

### Quantitation of leptin receptor protein levels in the hypothalamus of female mice

There is no difference in leptin receptor levels between female β2GPI-/- and diet matched female WT mice (data not shown) (p > 0.05).

### The effect of β2GPI deficiency on hypothalamus appetite signaling pathways in female mice

Intact Signal transducer and activator of transcription 3 (STAT3) signaling in the hypothalamus is an important regulator of food intake and has been implicated in mediating a role in leptin's anorectic functions [27]. Malonyl-CoA, an intermediate in fatty acid synthesis, serves as an indicator of energy status in the hypothalamic neurons [28]. Increased hypothalamic malonyl-CoA suppresses food intake [28]. The cellular malonyl-CoA level is determined by its rate of synthesis, catalyzed by Acc, and rate of removal by FAS [28]. Malonyl-CoA levels are also under the control of AMP kinase which phosphorylates/inactivates Acc [29]. Hypothalamic Acc activation is an important contributor to leptin's anorectic actions [30]. Inactivation of hypothalamic FAS protects mice from diet induced obesity [31].

**Figure 8:**
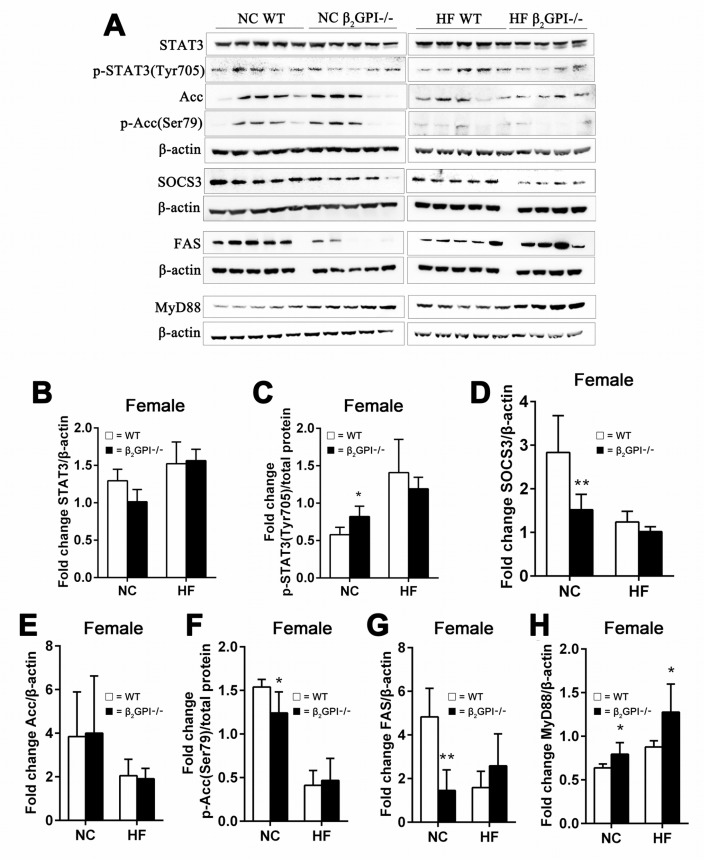
β2GPI deficiency and the hypothalamic appetite signaling pathways **(A)** Western blot analysis of hypothalamic proteins in female mice. (B-H) quantification of the immunoreactive bands **(B)** STAT3, **(C)** p-STAT3 (Tyr705) **(D)** SOCS3, **(E)** Acc, **(F)** p-Acc(Ser79), **(G)** FAS/β-actin and (H) MyD88. □ = WT, ■ = β2GPI-/-, NC = normal chow, HF = high fat. Data are expressed as mean ± SD (n = 4-5 mice per group). ^*^p < 0.05. ^**^p < 0.01.

We examined the total and phosphorylated levels of STAT3, Erk1/2, and Acc and the total level of FAS and Suppressor of cytokine signalling 3 (SOCS3) in the hypothalamus of female mice fed NC or HF diet at 16 weeks. In NC fed mice there was no difference between β2GPI-/- and WT mice in total STAT3 levels (Figure 8A, 8B). There was a statistically significant increase in p-STAT3 (Tyr705) in female β2GPI-/- compared to female WT mice fed NC (Figure 8A, 8C). SOCS3 an inhibitor of STAT3 [32] was significantly downregulated in the female β2GPI-/- mice fed a NC diet when compared to female WT mice fed a NC diet (Figure 8A, 8D). Hypothalamic Erk1/2 is a member of the MAPK family and is an additional downstream pathway of the leptin receptor, and plays a role in food intake, body weight and thermogenic sympathetic outflow [33]. Total and phosphorylated levels of Erk1/2 were not different in diet matched female β2GPI-/- and female WT mice (data not shown). In the female β2GPI-/- compared to female WT mice fed a NC diet there was a decrease in the level of phosphorylated Acc (Ser79) and a decrease in the total level of FAS (Figure 8A, 8E-8G). In contrast in the HF diet fed female mice, there was no significant difference in any of the described enzyme levels between WT and β2GPI-/- female mice (Figure 8A-8G).

Increased MyD88 signalling in the hypothalamus has been found to contribute to fatty acid induced leptin resistance and diet induced obesity [34]. In the female β2GPI-/- mice compared to the female WT mice, both for NC and HF diet fed mice, there is increased MyD88 in the hypothalamus (p < 0.05) (Figure 8A, 8H).

### There is no difference in glucose tolerance test in WT and β2GPI deficient mice fed a high fat diet

**Figure 9:**
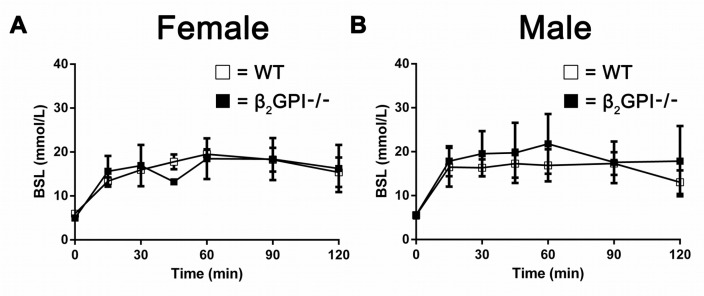
Effect of a glucose tolerance challenge on plasma glucose in WT and β2GPI deficient mice fed a high fat diet **(A)** Female mice. **(B)** Male mice. Blood glucose levels were measured over 125 min after intraperitoneal injection of glucose. □ = WT, ■ = β2GPI deficient mice.

Male and female WT and β2GPI deficient mice did not display any difference in blood glucose levels over 125 min following a glucose tolerance challenge test after overnight fasting (Figure 9A, 9B).

## DISCUSSION

In this paper we describe that female mice deficient in β2GPI are more prone to visceral fat accummulation compared to WT females when fed either a normal chow or high fat diet. Analysis of the visceral adipose tissue was consistent with a process of increased lipogenesis in the female β2GPI -/- mice fed either a normal or high fat diet diminished lipolysis in the female β2GPI -/- mice fed NC diet.

In the setting of being fed a normal chow diet, the female β2GPI -/- mice appear to have a compensatory decreased satiety response, driven by increased leptin levels, leading to increase in the anorexic signalling pathways of the hypothalamus including activation of STAT3. Female β2GPI -/- mice fed a high fat diet do not have a compensatory decrease in their satiety response. Despite an increase in visceral fat deposition, they consumed on a weekly basis an equivalent amount of high fat diet to their WT counterparts, and hence gained significantly more weight. The high fat diet fed female β2GPI -/- mice had a trend towards elevated leptin levels in their plasma, raising the possibility of them having increased resistance to the central anorexogenic actions of leptin in the hypothalamus. Though STAT3 and Acc signalling in the hypothalamus was equivalent between the female β2GPI deficient mice and the female WT mice fed a high fat diet, the former had increased levels of hypothalamic MyD88 which has been implicated as contributing to leptin resistance in the setting of mice been fed a high fat diet [34]. As a point of contrast, in male mice, fed either a normal chow or high fat diet, β2GPI deficiency does not influence weight gain, VAT accumulation and lipogenesis enzymes in the VAT compared to WT male controls. This indicates that β2GPI regulates fat metabolism in mice in a sexually dimorphic manner.

We noted WT females when fed a HF diet were protected from early onset obesity until 12 weeks of feeding. In male WT mice this early protective effect is not seen and the HF fed mice gain more weight than WT males fed NC within the first week. This phenomenon has previously been reported and as far as we are aware no mechanism has been described to explain this sexually dimorphic response [10, 35]. If we assume that weight gain is due to energy storage as reflected by lipogenesis activity, whilst lipolysis reflects fat breakdown, then our results showing down regulation of the lipogenesis proteins in the WT females fed a high fat diet when compared to WT females fed NC, suggest that WT females are protected from early onset obesity due to down regulation of the lipogenesis pathways. If the lipolysis pathways were contributing to this protective effect we would have expected to see their upregulation in WT females fed HF, whereas in contrast we saw that the lipolysis proteins were down regulated. Female β2GPI-/- mice fed a HF diet lost this early protective anti-obesity effect seen in WT females fed a HF diet, and they gained significantly more weight starting within the first week when fed a HF diet. They had a significant increase in the protein expression of the lipogenesis enzyme FAS and decreased phosphorylation of Acc (Ser79) compared to WT females fed a HF diet. This suggests that a mechanism by which β2GPI mediates its protective anti-obesity effect in females is by inhibiting the lipogenesis process from occurring in VAT. However, β2GPI also promotes lipolysis in VAT of females fed a NC diet and this may also contribute to its anti-obesity effect in female mice.

It has been noted in a previous study that exposure to a high fat diet for 12 weeks led to greater weight gain in C57BL/6J males compared to age matched females [35]. This study noted that inflammatory genes were up-regulated in males when compared to females, and there was a partial reversal after oophorectomy, where it was noted that weight gain and adipose tissue gene expression was more ‘male like’ [35]. It is therefore relevant to note that in our study, β2GPI deficient females, whether fed a normal chow or high fat diet had an increase in their intra-abdominal/gonadal tissue fat compared to WT females. Furthermore, β2GPI deficient females fed a high fat diet had an increase in their systemic inflammatory response as well as macrophage infiltration of VAT. These findings suggest that in the absence of β2GPI female mice acquire a more ‘male like’ fat distribution phenotype and an accompanying inflammatory response.

Recently it has been noted that β2GPI is able to attenuate lipopolysaccharide (LPS)/endotoxin induced signaling through Toll-like receptor 4 (TLR4) by a mechanism involving diverting the β2GPI-LPS complex to bind instead to low-density lipoprotein receptor-related protein 8 (cell surface receptor also known as ApoER2) [36], suggesting this receptor related mechanism may be involved in modulating fat storage in the β2GPI deficient compared to the wild-type female mice. Low grade endotoxemia has been well documented to contribute to the obesity promoting effects of a high fat diet through complex mechanisms [37, 38]. The finding of elevated CRP and macrophage infiltration in the VAT in female β2GPI -/- mice fed a high fat diet, as well as increased levels in the hypothalamus of MyD88 in NC and high fat diet fed female β2GPI -/- mice supports the possibility of increased gut derived LPS mediated systemic signaling through the TLR4/MyD88 pathway in the absence of β2GPI. The activation of the TLR4/MyD88 pathway disrupts the ability of adipose tissue known as ‘beige fat’ to switch towards brown fat, hence promoting the energy conserving, fat storage white adipose tissue phenotype [39]. Studies in rodents have shown that beige adipocytes are flexible being able to convert between brown and white phenotype [40]. Such regulatory signaling within beige fat has been implicated as being disrupted with the FTO obesity variant in humans, promoting a pro-obesity phenotype [4]. In this regard the recently described association between specific β2GPI and FTO SNPs conferring a benefit towards a healthy leanness phenotype is relevant [17]. We speculate β2GPI may promote leanness by maintaining the brown fat phenotype by inhibiting activation of the TLR4/MyD88 pathway in adipose tissue, though further study is necessary regarding this hypothesis.

In summary our findings support a role of β2GPI in preventing fat deposition in visceral fat and an accompanying systemic inflammatory response in females. The precise mechanism remains to be delineated.

## MATERIALS AND METHODS

### Animals and diet

C57BL/6J WT mice and β2GPI-/- mice on a C57BL/6J background were used in this study [41]. All animal experimental protocols were approved by the UNSW Animal Care and Ethics Committee. All mice were group housed in the animal house, St George Hospital, University of New South Wales, at 20 +/− 2°C on a 12/12-hour dark-light cycle and had ad libitum access to water and food. Eight-week-old animals (n = 5) for each genotype, diet and sex were randomly allocated into 8 groups (female WT, NC and HF; female β2GPI-/- NC and HF; male WT, NC and HF; male β2GPI-/- NC and HF fed animals) in order to test the influence of genotype, diet and sex on weight gain. NC, was from Gordon's Specialty Stockfeeds, Yanderra, NSW, Australia while the HF, SF00-219 was from Specialty Feeds, Glen Forest, Western Australia, Australia and contained 19.4 kJ/g; energy 40% fat, 17% protein, 43% carbohydrate. The mice were weighed weekly and food intake was measured initially then weekly by monitoring the weight of the remaining food and the mice were sacrificed after 16 weeks of dietary treatment. At the time of sacrifice mice were fasted overnight for 10 - 14 h and anesthetized by isoflurane inhalation.

### Sample collection

Blood samples were collected by cardiac puncture in 1 mL tubes containing K3 EDTA (Greiner Vacuette Minicollect K3 EDTA tube; Greiner Bio-one GmbH, Kremsmünster, Austria). They were centrifuged (1,000 g for 10 min) and the subsequent plasma fraction collected and stored at −80°C until use. Female and male, WT and β2GPI-/- mice were perfused with phosphate-buffered saline (PBS) through the left ventricle following cardiac puncture for blood collection. After the female mice were euthanized, the whole hypothalamus was surgically excised and snap frozen in liquid nitrogen. Female and male WT and β2GPI-/- mice fed NC and HF diets had their VAT (parametrial/epididymal) removed, weighed and divided in three parts, one was added to RNA stabilisation agent (Qiagen, Hilden, Germany), one was directly snap frozen and both were stored in liquid nitrogen at -80°C and the third portion was fixed in 10% neutral buffered formalin until analysed.

### ELISA assays

ELISAs for quantitating leptin were from Merck Millipore (Bedford, MA) and CRP was from (R&D Systems, Minneapolis, MN). Levels were quantitated following the manufacturer's instructions.

### RNA isolation, cDNA synthesis, and qPCR

Total RNA from mouse VAT specimens was extracted with the RNeasy Lipid Tissue Mini Kit from Qiagen, (Hilden, Germany) according to the manufacturer's instructions. cDNA was obtained by reverse transcribing the total RNA with a QuantiTect Reverse Transcription Kit (Qiagen). Amplification and analysis were performed on the Bio-Rad CFX96TM Real-Time PCR System (Bio-Rad, Hercules, CA). The primers were from Qiagen for all target genes. GAPDH was used as an internal control, and target gene values were normalized to those of GAPDH. Relative mRNA expression levels were calculated using a comparative ΔΔCt method. ΔCt (experimental) = Ct (target gene) - Ct (GAPDH), ΔCt (control) = Ct (target gene) - Ct (GAPDH), ΔΔCt = ΔCt (experimental) -ΔCt (control), then the fold-change in gene expression was derived by 2-ΔΔCt.

### Quantitation of proteins in VAT and hypothalamus by western blotting

VAT was homogenised in radioimmunoprecipitation assay buffer containing 1% protease and 1% phosphatase inhibitor cocktails (Sigma-Aldrich, St Louis, MO) centrifuged at 12,000 g for 15 min, and the supernatant was harvested while carefully avoiding the lipid layer on top. Protein concentration was measured with a BCA protein quantification kit (Thermo Fisher Scientific, San Jose, CA). Protein extracts were separated on a 4–12% NuPAGE gel (Thermo Fisher Scientific) and then transferred onto Immobilon FL PVDF membrane (Merck Millipore). Membranes were blocked with 5% BSA or skim milk at RT for 1 h and incubated with primary antibodies either FAS, Acc, p-Acc (Ser79), Atpcl, p-Atpcl (Ser455), STAT3, p-STAT3 (Tyr705), SOCS3, Erk1/2, p-Erk1/2 (Thr202/Tyr204), Hsl, p-Hsl (Ser563, Ser660, Ser565), all were from Cell Signaling Technology (Beverly, MA). Antibodies to MyD88 and leptin receptor were from (Abcam, Cambridge, UK) and an antibody to β-actin was from (Sigma-Aldrich). After three consecutive 5 min washes in Tris-buffered saline-Tween 20 (TBS-T) (0.1%) (Sigma-Aldrich), membranes were incubated with rabbit/mouse HRP–conjugated secondary antibodies (DAKO; Glostrup, Denmark) for 1 h at RT. After three further washes in TBS-T, ECL reagent was added (GE Healthcare, Milwaukee, MI) and incubated for 1 min. The membranes were scanned and the band density quantified with the Image analysis system-ImageQuant LAS4010 and Image Quant software (GE Healthcare, Milwaukee, MI). The relative expression of target = the grey value of target protein/grey value of the housekeeping gene β-actin. Membranes were stripped with the stripping agent Restore (Thermo Fisher Scientific) according to the protocol of the manufacturer.

### Histology and histomorphometry

Adipose tissues were isolated from either epididymal or parametrial areas and fixed in 10% neutral buffered formalin. The tissues were then processed for paraffin embedding. Multiple 6 μm-thick microtome sections from each tissue were stained with H&E and photographed. For measurement of adipocyte area, 200 adipocytes from each VAT sample were sampled and the area for each adipocyte was calculated and expressed as a mean for each mouse.

### Immunohistochemistry

For immunohistochemical studies, 3-μm sections of VAT samples were cut from each specimen and mounted on silane-coated slides. An anti-MAC-2 antibody was from (Cedarlane, Burlington, Canada) and used for macrophage immunolabelling using the streptavidin-biotin-peroxidase complex method, with a commercial detection system (DAKO) following the manufacturer's instructions. Antigen retrieval was by microwave treatment in citrate buffer (pH 6.0) three times for 5 min each in a 750 W microwave oven, followed by cooling for 20 min at RT. The primary antibody MAC-2 was diluted 1 in 1000 in PBS and applied to the VAT samples for 1 h at RT. The immunoreaction was visualized by incubation with 3, 3′-diaminobenzidine tetrahydrochloride (DAB) at 0.05% with 0.01% H_2_O_2_ as the final substrate for 5 min. After a final washing in distilled water, the sections were counterstained with haematoxylin, dehydrated, cleared and mounted. The primary antibody was replaced by normal goat IgG (R&D Systems) as negative controls. Positive controls consisted of WT mouse liver tissue for MAC-2.

### Glucose tolerance challenge tests

In selected mice (n = 4 to 5) at the end of the study an intraperitoneal tolerance test was performed in overnight fasted mice as previously described my McGrath et al [42].

### Statistical analyses

Statistical analyses were performed using the Mann–Whitney test. All analyses performed used the GraphPad Prism version 5.01 for Windows (GraphPad Software, La Jolla, California, www.graphpad.com). P < 0.05 were considered statistically significant.

## Abbreviations

Acc: acetyl-CoA carboxylase
AMPK: AMP-activated protein kinase
ApoH: apolipoprotein H
Atcpl: ATP citrate lyase
β2GPI: β2-glycoprotein I
BMI: Body Mass Index
CRP: C reactive protein
FAS: fatty acid synthase
FTO: Fat Mass and Obesity Associated variant
HF: high fat diet
Hsl: hormone-sensitive lipase
NC: normal chow diet
p-Atpcl: phospho-ATP citrate lyase
PKA: activated protein kinase A
Ser: serine
SOCS3: suppressor of cytokine signalling 3
STAT3: signal transducer and activator of transcription 3
VAT: visceral adipose tissue
WT: wild type
